# ﻿New species and infrageneric names in *Poa* L. (Poaceae): transferring species of *Agrostopoa* Davidse, Soreng & P.M. Peterson and *Raimundochloa* A.M.Molina and some notes on misidentifications

**DOI:** 10.3897/phytokeys.266.173731

**Published:** 2025-11-10

**Authors:** Robert J. Soreng, Lynn J. Gillespie

**Affiliations:** 1 Department of Botany, National Museum of Natural History, Smithsonian Institution, Washington 20013-7012 DC, USA National Museum of Natural History, Smithsonian Institution Washington United States of America; 2 Botany Section, Research and Collections, Canadian Museum of Nature, Ottawa K1P 6P4 ON, Canada Canadian Museum of Nature Ottawa Canada

**Keywords:** *
Agrostopoa
*, classification, DNA, GPWG III, *
Poa
*, PPAM, *
Raimundochloa
*, *
Rostraria
*, taxonomy

## Abstract

Based on DNA studies, the species of *Agrostopoa* Davidse, Soreng & P.M. Peterson and *Raimundochloa* A.M.Molina are here transferred to *Poa* L.: *Poa barclayae*, *P. wallisii*, *P. woodii*, **comb. nov.** and *P. ana-molinae***nom. nov.***Raimundochloa trachyantha* (Phil.) A.M.Molina is placed in P. subg. Parodiochloa (Soreng) Soreng & L.J.Gillespie, stat nov. In addition, we correct or comment on phylogenetic reports of species of *Bellardiochloa* Chiov., *Nicoraepoa* Soreng & L.J.Gillespie and *Cutandia* Willk., aligning in *Poa*.

## ﻿Introduction

A little house-keeping is needed to add four species to *Poa* from two genera (*Agrostopoa* Davidse, Soreng & P.M. Peterson and *Raimundochloa* A.M.Molina) previously placed in synonymy of *Poa* L. (Soreng et al. 2017, 2022a), bringing the genus total to 612 accepted species (by Soreng’s accounting of May 2025). We also account for a few DNA vouchers misidentified in literature as resolving within *Poa* and one that needs further study. Herbarium acronyms follow Thiers (2025).

### ﻿A transfer of the three species of *Agrostopoa* to *Poa*

*Agrostopoa* was described by Davidse et al. (2009) with three species, one annual and two perennials, from paramo and superparamos of northern Colombia’s Sierra Nevada de Santa Marta and Sierra Nevada del Cocuy, between 3450 and 4450 m elevation. *Agrostopoa* was diagnosed as differing from *Poa* by 1-flowered spikelets, lemma awned or mucronate and characterised as having panicle branches spreading, sinuous, mostly delicate, fragile and smooth, lemmas keeled and glabrous, palea keels smooth or slightly scabrous, anthers 3 (rarely 2). The species are rarely collected, only four collections being known to us at that time and little is known about them. Based on morphology alone, the enigmatic genus was placed in subtribe Poinae Dumort. [Poinae circumscribed at that time as subset of genera within the PPAM clade, aligning within *Poa* as identified by plastid DNA and nrDNA (Gillespie et al. 2008): *Anthochloa* Nees & Meyen, *Aphanelytrum* (Hack.) Hack., *Austrofestuca* (Tzvelev) E.B.Alexeev, *Dissanthelium* Trin., *Eremopoa* Roshev., *Neuropoa* Clayton, *Tovarochloa* T.D.Macfarl. & But and *Tzvelevia* E.B.Alexeev [and *Arctopoa* (Griseb.) Prob. for plastid data, but not nrDNA] or near *Poa*, including subtribe Cinninae, but excluding Alopecurinae, Miliinae or Puccinelliinae (now = Coleanthinae)]. Soreng et al. (2015) expanded Poinae to also include Alopecurinae and placed all of the above genera in *Poa*, except for the reticulate genus *Arctopoa*. Soreng et al. (2017, 2022a) narrowed Poinae to the genus *Poa* and tentatively placed *Agrostopoa* in subtribe Cinninae.

The Grass Phylogeny Working Group III (2024 [GPWG III]) generated the first published nuclear (ERR7621071) and plastome DNA sequences for any of the species, from *J.R.I. Wood 5268* (K), an isotype of *A. woodii* Davidse, Soreng & P.M. Peterson. The GPWG III analyses placed that species in *Poa*, but in an ambiguous relationship within a large clade of *Poa*, sister to P. subg. Sylvestres (Soreng) Soreng & L.J.Gillespie, above *Poa annua* L. in plastid tree, but not among representatives of subg. Poa, nor of subg. Stenopoa (Dumort.) Soreng & L.J.Gillespie. Until this new data can be meshed with more taxa of *Poa*, the infrageneric placement of *Agrostopoa* with that dataset will remain uncertain. An IQtree analysis of unpublished ITS data from the *A. woodii* holotype, sequences generated by K. Romaschenko in September 2018, clearly places the species within a large mostly unresolved polytomy set of **h**-clade species in Gillespie’s large matrix of *Poa* (Romaschenko pers. comm. 2025; see Soreng et al. (2020) for clade coding). We note that the one species of P. subg. Poa sect. Tovarochloa (T.D. Macfarl. & But) Molinari, *P. apiculata* Refulio, a rare and tiny annual of the Peruvian Andes, also has one floret per spikelet and apiculate lemmas, reminiscent of *Agrostopoa*. That species aligns with some other South American oddities [*Dissanthelium* Trin. (pro parte typica) and *Aphanelytrum* (Hack.) Hack.], on a long branch within the **H**-clade in both plastid and nrDNA analyses. Taxonomically, this is Poa subg. Poa supersect. Homalopoa Dumort. We hypothesise that the founder populations of more normal looking *Poa* mutated extensively through inbreeding leading to the odd morphologies and long phylogenetic branches in this subset of taxa.

#### ﻿ Poa barclayae

Taxon classificationPlantaePoalesPoaceae

(Davidse, Soreng & P.M. Peterson) Soreng comb. nov.

C7F458E5-C1DF-536D-AE59-33136A1A230C

urn:lsid:ipni.org:names:77371718-1

Agrostopoa barclayae Davidse, Soreng & P.M. Peterson, Novon 19(1): 36, 38, f. 2. 2009: Basionym. Type: Colombia: Magdalena: Sierra Nevada de Santa Marta, alrededores de cabeceras de Rí: Sevilla, 3490 m elev., 20 Jan 1959, *H.G. Barclay & P. Juajibioy 6567* (holotype: MO5114991; isotypes: COL, K, US01049749).

#### ﻿ Poa wallisii

Taxon classificationPlantaePoalesPoaceae

(Mez) Soreng comb. nov.

18592F23-71B4-5107-B1AE-715411E5E76B

urn:lsid:ipni.org:names:77371719-1

Muhlenbergia wallisii Mez, Repert. Spec. Nov. Regni Veg. 17(13–18): 214. 1921: Basionym. Type: Colombia: Sierra Nevada de Santa Marta, *G. Wallis* [lectotype (designated by Davidse et al. (2009, p. 34)): US-90978 (fragm. ex B); isolectotype: US-90979 (fragm. ex B)].
≡ Agrostopoa wallisii (Mez) P.M. Peterson, Soreng & Davidse Novon 19(1): 34, 36, f. 1. 2009.

#### ﻿ Poa woodii

Taxon classificationPlantaePoalesPoaceae

(Davidse, Soreng & P.M. Peterson) Soreng comb. nov.

D345A11D-B938-5FCB-A665-CD3A9EDE1BBF

urn:lsid:ipni.org:names:77371720-1

Agrostopoa woodii Soreng, P.M. Peterson & Davidse Novon 19(1): 38, f. 3. 2009: Basionym. Type: Colombia: Boyacá: Sierra Nevada del Cocuy, Boquerón cf. Cusiri, 4450 m elev., 31 Dec 1985, *J.R.I. Wood 5268* (holotype: US01049750; isotype: COL000359964, K000975022).

### ﻿*Poa ana-molinae*

Ana Maria Molina (1986a) published a new monotypic genus *Parodiochloa* A.M. Molina (type *Koeleria trachyantha* Phil.). The new genus turned out to be a later homonym of *Parodiochloa* C.E. Hubbard, leading Molina (1986b) to rename her genus as *Raimundochloa* A.M. Molina. Based on the invalid combination of *Rostraria trachyantha* (Phil.) Tzvelev (Tzvelev 1976. p. 267, without basionym citation), annual habit and morphological similarities with other *Rostraria* species, including rather rough lemma texture and presence of awns, Soreng (2003) validated the combination *Rostraria trachyantha* (Phil.) Tzvelev ex Soreng, placing *Raimundochloa* as a synonym of that genus. With the first DNA data for the species (Persson and Rydin 2016) and their result confirmed for a second sample (Gillespie data of 2017), *Raimundochloa* was placed in synonymy of *Poa* by Soreng et al. (2017, 2022a). Here we transfer the single species to *Poa* where a new name is required.

#### ﻿ Poa ana-molinae

Taxon classificationPlantaePoalesPoaceae

Soreng & L.J. Gillespie nom. nov.

E5543EDB-71D1-5FDA-B144-180000FC5F9E

urn:lsid:ipni.org:names:77371721-1

Koeleria trachyantha Phil., Fl. Atacam. 55 (N^o.^ 404). 1860: Basionym. Type: [lectotype (here designated by V. Finot, G. Rojas & Soreng): SGO-O63517; isolectotype: BAA (fragm. ex SGO-63517), US-(SGO-PHIL-230 photo “Trisetum brachyantherum Phil.”), W-01160039938 (ex herb. Hackel, ex SGO-PHIL-230, “Trisetum brachyantherum Ph.”), W0028853 (ex SGO-230 or 63517?)]. Type Protologue: Chile, Prov. de Antofagasta, Paposo, Nov 1853, *Philippi s.n.*
≡ Parodiochloa trachyantha (Phil.) A.M. Molina, Parodiana 4(1): 112–120, f. 1–2. 1986. ≡ Raimundochloa trachyantha (Phil.) A.M. Molina, Parodiana 4(2): 402. 1986. ≡ Rostraria trachyantha (Phil.) Tzvelev ex Soreng, Contr. U.S. Natl. Herb. 48: 604, 4. 2003. (non-Poa trachyantha Hack., 1912.).

##### Note.

The species is here renamed in honour of Dra. Ana María Molina, Argentine Botanist, Agrostologist.

According to Finot (Finot 2022, p. 966), this unusual annual species occurs in the coastal Loma vegetation of the Atacama Desert in northern Chile and southern Peru (24°50'–33°39'S), has peculiar rough upper glume and lemma surface texture “escabroso-hirsutas, coriaceous” and tightly contracted panicles similar to *Rostraria cristata* (L.) Trin. and lemmas with short apical awns (1–2 mm), the lemma apex sometimes apically short lobed (see Illustration, in Finot 2022, p. 965, Lámina 357). These characteristics are reminiscent of the densely scabrous and coriaceous lemmas with stiff apical awns in *Poa flabellata* (Lam.) Raspail (the type species of *Parodiochloa* C.E.Hubb., also a synonym of *Poa*). Curiously both of these species have short, stiff apical awns on the lemmas (rare in *Poa*) and narrow, congested panicles (extreme in *P. ana-molinae* for *Poa*).

Plastid and nrDNA data place *Poa ana-molinae* in well supported and consistent clades named “Parodiochloa” (**R** plastid and **r** nrDNA clades; see Soreng et al. (2020) for clade coding), with formally named sections Poa sect. Parodiochloa (C.E.Hubb.) Soreng and P. sect. Tzvelevia (E.B.Alexeev) Soreng & L.J.Gillespie. The **Rr**-clade hitherto was demonstrated to contain a series of perennial species with narrow, compact, contracted panicles, from widely distributed sub-Antarctic oceanic islands and Tierra del Fuego [*P. cookii* (Hook.f) Hook.f, *P. flabellata*, *P. kerguelensis* (Hook.f.) Steud. (type of *Tzvelevia* ≡ Poa sect. Tzvelevia), *P. ramosissima* Hook.f.] and one annual species with open panicles from south-eastern United States, *P. chapmaniana* Scribn. (Gillespie et al. 2007; Refulio-Rodríguez et al. 2012; Persson and Rydin 2016; Soreng et al. 2022b). Both of the annuals, *P. ana-molinae* and *P. chapmaniana* (type of P. sect. Diversipoa Chrtek & V. Jirásek) have one stamen per flower, but later has a web on the callus and lemmas with well-developed soft pubescence on the keel and marginal nerves and shorter hairs on the surface and open, pyramidal, thin-branched panicles, while the other taxa in the Parodiochloa clade (including *P. ana-molinae*) lack callus hairs and lack much (at most, coarse and short) pubescence on their lemmas and have contracted panicles. The three sections are united here in a subgenus Parodiochloa. The two sections mentioned above were formerly placed in P. subg. Ochlopoa (Asch. & Graben) Hyl. However, the monophylly of the Ochlopoa clade as originally resolved and constituted (Soreng and Gillespie 2007; Gillespie et al. 2008) no longer has support (Soreng et al. 2020) in nrDNA trees and we here restrict subg. Ochlopoa to encompass species included in P. sect. Micrantherae Stapf. (type *P. annua* L.), including *P. cyrenaica* A. Durand & Barratte (formerly the sole species in the genus *Libyella* Pamp.).

### ﻿Poa subg. Parodiochloa

#### ﻿ Poa subg. Parodiochloa

Taxon classificationPlantaePoalesPoaceae

(C.E.Hubb.) Soreng & L.J.Gillespie, comb. et stat. nov.

70E612A6-43EE-5D43-B120-A019A60A9F86

urn:lsid:ipni.org:names:77371722-1

Parodiochloa C.E.Hubb., Brit. Mus. (Nat. Hist.), Bot. 8: 395. 1981. Type: Festuca flabellata Lam. ≡ Poa flabellata (Lam.) Raspail
≡ Poa sect. Parodiochloa (C.E.Hubb.) Soreng, Contr. U.S. Natl. Herb. 48: 579. 2003.
= Parodiochloa A. M. Molina (1986a), nom. illeg. hom. ≡ Raimundochloa A.M.Molina (1986b).Type: Koerleria trachyantha Phil (≡ Poa ana-molinae).

##### Sections included.

Poa sect. Diversipoa, P. sect. Monandropoa Parodi (tentatively), P. sect. Parodiochloa, P. sect. Tzvelevia.

*Poa chapmaniana* and *P. ana-molinae* might be placed together in P. subg. Parodiochloa sect. Diversipoa. However, we wonder if the former might have arisen via hybridisation with other *Poa* species in North America like the annual *P. bigelovii* Vasey & Scribn. (originally included in Poa sect. Diversipoa (Chrtek and Jirásek 1962) along with other North American annuals, all **Hph** types), which has similar lemma pubescence and infrequent reduction to 1 or 2 stamens where it approaches the geographic range of *P. chapmaniana* or even older hybridisations in Latin America (Soreng 2007).

What is really going on will hopefully become apparent once more nuclear DNA of more species of *Poa* become available. We wonder if species with **Rr** genotypes were once more diverse and widespread, but continental American species nrDNA signals were mostly swamped out by concerted evolution and their chloroplasts displaced by in hybridisation with more numerous northern and southern Western Hemisphere species of Poa supersect. Homalopoa.

One stamen is rare in *Poa*, but *P. tucumana* Parodi, of the monotypic P. sect. Monandropoa (Parodi 1962), also has one anther per floret. The species is known from the Argentinian Provinces Tucuman and Catamarca (Giussani et al. 2012) and, here, it is tentatively placed in P. subg. Parodiochloa. A recent collection from the next province north (Prov. Salta; E slope of Sierra de Santa Victoria, 12 air km ESE of Abra Lizoite on new road to Santa Victoria, 4175 m elev., margins of a small alpine tarn, 22.26850S, 65.11736W, 29 Mar 2006, *Peterson et al. 19606* US [voucher misplaced at US]), with its perennial habit, small stature and open panicles might belong to this species (RJS recall). This accession aligns within the Parodiochloa clade in both plastid and nrDNA trees. *Poa tucumana* is small, slender perennial species exhibiting open panicles, small, 2-flowered spikelets, glumes 3-nerved, floret callus with soft, short (to 2 mm long) hairs, callus with woolly hairs surrounding the lemma base, lemmas 5-nerved, unawned, keel scaberulous, lower body with a few minute appressed hairs, anthers one per flower, similar in some respects to *P. chapmaniana* (Giussani et al. 2012). Its slender unbranched stigmas, although vestigial, are reminiscent of the linear styles of *P. flabellata*.

## ﻿Discussion of a few sequenced collections putatively representing other genera resolved within *Poa*

It is critical to prepare or have vouchers for new DNA sequences and to have vouchers checked by specialists, particularly when growing plants from seed repositories. Sequences of *Bellardiochloa* Chiov. and *Nicoraepoa* Soreng & L.J.Gillespie, grown from USDA Plant Introduction Station seed (PI numbers), were resolved within *Poa* (Hackel et al. (2018), supplementary tree S2; Orton et al. (2021)). The samples were initially used for the GPWG III analyses, but were rejected by Soreng. Two other genomic samples purportedly species of *Festuca* and *Cutandia* that resolved in *Poa* in recent studies are discussed.

Plants grown from seed of PI 442548, “*Bellardiochloa variegata* (Lam.) Kerguelen”, yielded complete a plastid genome (Orton et al. (2021); MT119358 and NC_059979.1), which resolved within *Poa* with *P. trivialis* L. (complete plastid genome) and are most likely from seed contaminated with that. Although sometimes placed in *Poa* (Clayton and Renvoize 1986), *Bellardiochloa*, as clearly shown by morphology and numerous DNA studies (Soreng 1990; Soreng and Davis 2000; Gillespie et al. 2007, 2008, 2010, 2022b; Cabi et al. 2015) does not belong in *Poa.* Another PI 253455 seed accession, originally reposited as *Poa* sp., was re-identified by RJS as *Bellardiochloa violacea* (Bellardi) Chiov. and used as an outgroup for a plastid DNA restriction site study of *Poa* (Soreng 1990). By priority, the name is correctly *B. variegata*. Cabi et al. (2015) conducted a phylogenetic analysis of five species of the genus *Bellardiochloa*, which is now placed in Poeae subtribe Ventenatinae Holub ex L.J. Gillespie, Cabi & Soreng (Soreng et al. 2017, 2022a). The genus differs from *Poa* by, among other characters, lemmas rounded on back, glabrous and terminated by a brief awn, rachilla internode-floret callus break points rimmed by a short crown of hairs, caryopsis semi-soft (versus hard in *Poa*).

“*Nicoraepoa erinacea* (Speg.) Soreng & L.J. Gillespie”, seed grown from PI 204263, yielded complete plastid genomes (Orton et al. (2021); MT094317 {as *Poa* sp.}, NC_058910.1 {as *N. erinacea*}), the sequences being identical. Seed of this accession was recently regrown in Beltsville USDA by Melanie Schori in 2025, and the flowering voucher was determined by RJS in 2025 to be *Poa palustris* L. *Nicoraepoa erinacea* is a rare species of Patagonian hyper-saline canyons in western Chubut Prov., Argentina. The source of the original PI collection (*A.A.Beetle*; 416/52; Argentina; Chubut; Quichaura Ranch, near Tecka, ca. 100 kil. s. of Esquel. US 2076560 Barcode: 04053281) is authentic, but the regenerated seed became contaminated. The genus *Nicoraepoa* (6 spp. of subtribe Hookerochloinae Soreng & L.J. Gillespie) was named and investigated by Soreng and Gillespie (2007) using plastid sequences (Gillespie et al. 2007). Subsequently, Gillespie et al. (2010) demonstrated that one of the species, *N. pugionifolia* (Speg.) Soreng & Gillespie, also a species of hyper-saline wet cushions (*mallones*) in Patagonia, was reticulate between *Poa flabellata* (P. sect. Parodiochloa) (with which it shared nrDNA) and *Nicoraepoa* (probably *N. robusta*). Only one chromosome count is known for the genus, reported for *N. chonotica* (Phil.) Soreng & L.J. Gillespie [now N. andina subsp. chonotica (Phil.) Finot, Soreng & Giussani], 2*n* = 42 (Probatova et al. 2009). *Nicoraepoa erinacea* is most similar to *N. pugionifolia*, being of a more compact and indurate form. It might be of similar reticulate origin or the *Nicoraepoa* parent of the latter. The species warrants further study. Another PAFTOL sample of *Nicoraepoa*, *N. subenervis* (Hack.) Soreng & L.J. Gillespie, was likewise rejected by RJS in 2021, possibly contaminated with P. (sect. Stenopoa) palustris L. Plastid and nrDNA sequences of N. subenervis subsp. subenervis and subsp. spegazziniana (Nicora) Soreng & L.J. Gillespie align within *Nicoraepoa* (Gillespie et al. 2010).

“*Festuca pseudovina* Hack.” leaf material (PI 374046, originally from Hungary), was used to generate a plastid sequence MH569085 in Hackel et al. (2018), as “Poa subg. Stenopoa” in S2 tree), used by Orton et al. (2019, 2021), but no voucher was prepared. That sequence resolved as sister to a clade with *P. palustris* [two samples: *Saarella & Percy 1080*, and PI 204263 (identified above)] and *P. nemoralis* and we presume the seed sample was contaminated with a species of P. sect. Stenopoa. ShotGun nuclear DNA sequences used by GPWG III as “Poa sect. Stenopoa”) resolved the same sample in *Poa* with P. (sect. Stenopoa) attenuata Trin. (transcriptome data, from China, identification not verified), in a clade with *P. palustris* and we presume the seed sample was contaminated with a species of P. sect. Stenopoa. In 2020, Melanie Schorie tried to grow the seed at USDA ARS greenhouses in Beltsville, but nothing germinated.

*Cutandia stenostachya* (Boiss.) Stace, PAFTOL nuclear DNA voucher (*R.M. Burton s.n.*, K, K001418218), (Angiosperm 353 data ERR7621429), was pointed out by RJS in 2021 as a likely error, possibly for *Poa palustris* L., but it remained in the GPW III study and remains in Kew’s Tree of Life. The genus *Cutandia* (6 spp.) belongs to Poeae subtribe Parapholinae (or Cynosurinae s.l., sensu Tkach et al. (2024)), which includes a series of annual genera (Soreng et Davis (2000) - first DNA data for the genus; Soreng et al. (2022a)). Two other species of *Cutandia*, including its type species, have been sequenced and resolve within the subtribe. Stace (1978, 1985) accepted *C. stenostachya* in that genus. The species *C. stenostachya* is a bit odd in that genus as its branches do not disarticulate also the rachilla internodes appear to be terete (and quite elongated; Stace (1985), fig. 21), more like *Poa* and relatives than subtribes Loliinae and Parapholinae where rachilla internodes tend to be dorsoventrally compressed and have annulated floret calluses. However, as stranger things have happened, this taxon warrants further investigation.

## Supplementary Material

XML Treatment for Poa barclayae

XML Treatment for Poa wallisii

XML Treatment for Poa woodii

XML Treatment for Poa ana-molinae

XML Treatment for Poa subg. Parodiochloa

## ﻿Additional information

### Use of AI

No use of AI was reported.

### Author ORCIDs

Robert J. Soreng https://orcid.org/0000-0002-8358-4915

Lynn J. Gillespie https://orcid.org/0000-0003-3129-434X

### Data availability

All of the data that support the findings of this study are available in the main text.
